# Early rehabilitation using a passive cycle ergometer on muscle morphology in mechanically ventilated critically ill patients in the Intensive Care Unit (MoVe-ICU study): study protocol for a randomized controlled trial

**DOI:** 10.1186/s13063-015-0914-8

**Published:** 2015-08-28

**Authors:** Laura Jurema dos Santos, Fernando de Aguiar Lemos, Tanara Bianchi, Amanda Sachetti, Ana Maria Dall’ Acqua, Wagner da Silva Naue, Alexandre Simões Dias, Silvia Regina Rios Vieira

**Affiliations:** Graduate Program in Health Sciences: Cardiology and Cardiovascular Science, Universidade Federal do Rio Grande do Sul (UFRGS), Rua Dr Pereira Neto, 725 torre 1 ap 902, Tristeza–Porto Alegre, RS CEP 91920530 Brazil; Department of Physical Therapy, Universidade Luterana do Brasil (ULBRA), Canoas, RS Brazil; Graduate Program in Human Movement Science, Universidade Federal do Rio Grande do Sul (UFRGS), Porto Alegre, RS Brazil; Graduate Program in Respiratory Science, Universidade Federal do Rio Grande do Sul (UFRGS), Porto Alegre, RS Brazil; Physical Therapy Service–Intensive Care Unit, Hospital de Clínicas de Porto Alegre (HCPA), Porto Alegre, RS Brazil; Department of Physical Therapy, Universidade Federal do Rio Grande do Sul (UFRGS); Physical Therapy Service, Hospital de Clínicas de Porto Alegre (HCPA), Porto Alegre, RS Brazil; School of Medicine, Universidade Federal do Rio Grande do Sul (UFRGS); Intensive Care Unit, Hospital de Clínicas de Porto Alegre (HCPA), Porto Alegre, RS Brazil

**Keywords:** Intensive care, Early ambulation, Clinical trial

## Abstract

**Background:**

Patients in Intensive Care Units (ICU) are often exposed to prolonged immobilization which, in turn, plays an important role in neuromuscular complications. Exercise with a cycle ergometer is a treatment option that can be used to improve the rehabilitation of patients on mechanical ventilation (MV) in order to minimize the harmful effects of immobility.

**Methods/Design:**

A single-blind randomized controlled trial (the MoVe ICU study) will be conducted to evaluate and compare the effects of early rehabilitation using a bedside cycle ergometer with conventional physical therapy on the muscle morphology of the knee extensors and diaphragm in critical ill patients receiving MV. A total of 28 adult patients will be recruited for this study from among those admitted to the intensive care department at the Hospital de Clínicas de Porto Alegre. Eligible patients will be treated with MV from a period of 24 to 48 h, will have spent maximum of 1 week in hospital and will not exhibit any characteristics restricting lower extremity mobility. These subjects will be randomized to receive either conventional physiotherapy or conventional physiotherapy with an additional cycle ergometer intervention. The intervention will be administered passively for 20 min, at 20 revolutions per minute (rpm), once per day, 7 days a week, throughout the time the patients remain on MV. Outcomes will be cross-sectional quadriceps thickness, length of fascicle, pennation angle of fascicles, thickness of vastus lateralis muscle, diaphragm thickness and excursion of critical ICU patients on MV measured with ultrasound.

**Discussion:**

The MoVe-ICU study will be the first randomized controlled trial to test the hypothesis that early rehabilitation with a passive cycle ergometer can preserve the morphology of knee extensors and diaphragm in critical patients on MV in ICUs.

**Trial registration:**

NCT02300662 (25 November 2014).

## Background

Patients in Intensive Care Units (ICU) are often exposed to prolonged immobilization, which can play an important role in neuromuscular complications [1, 2]. Bed rest results in skeletal muscle weakness, progressing to muscle atrophy and losses of 3–11 % of muscle mass during the first 3 weeks of immobility [3]. This loss of muscle mass and muscle weakness, in turn, are the result of acquired myopathy, polyneuropathy or a combination of the two [4]. The prevalence of acquired polyneuropathy among patients in intensive care settings is in the range of 58–96 % [5]. Notwithstanding, recent evidence suggests that muscle weakness can be present within hours of starting mechanical ventilation (MV) and is evident in 25–100 % of patients ventilated for more than 7 days [6]. Among these patients, muscle weakness is associated with increased length of hospital stay and higher mortality and with impaired functional status that can still be detected years after hospital discharge, compromising patients’ quality of life [7, 8].

The combination of prolonged MV and the effects of immobility causes significant changes to muscle fibers, reducing both respiratory and peripheral muscle strength [9]. While this muscle weakness has a multifactorial etiology, early rehabilitation of patients in ICUs can help to keep the atrophy, loss of muscle mass and loss of physical conditioning associated with bed rest to a minimum [2]. One resource that has proved to be of great utility in hospitals is the cycle ergometer, which is a stationary piece of equipment designed to enable cyclical rotations of lower and/or upper extremities and can be used to perform passive, active and resisted exercises [1].

Treatment with the cycle ergometer has been shown to improve quadriceps strength, functional status and 6-min walking results at hospital discharge [10]. Although this therapy is widely used in outpatient settings with the objective of improving rehabilitation of patients with chronic pulmonary disease, few studies have assessed its effects in hospital settings, particularly in ICUs [11, 12].

This article provides a detailed description of the background, target population and methodology of the MoVe-ICU study, an randomized controlled trial (RCT) investigating the effects of early rehabilitation using a bedside cycle ergometer combined with conventional physical therapy versus conventional physical therapy alone on the muscle morphology of the knee extensors and the diaphragm.

## Methods

This study will be financed by the Fundação de Amparo à Pesquisa do Estado do Rio Grande do Sul (FAPERGS) research funding agency and it will be conducted in accordance with the principles laid out in the Helsinki Declaration and with Good Clinical Practice. Procedures will be in accordance with National Health Council (Conselho Nacional de Saúde: resolution number 466/12). The HCPA Ethics Committee has approved the study (CEP HCPA number 10-0530) and informed consent will be obtained in writing from all patients who are taking part.

### Patients

This is a single-blind RCT that is going to be conducted by the intensive care and physiotherapy departments at the Hospital de Clínicas de Porto Alegre (HCPA)–Universidade Federal do Rio Grande do Sul (UFRGS). Patients of both genders aged ≥18 years will be recruited from among those admitted to the HCPA ICU and undergoing MV from 24 to 48 h after transfer from the emergency department or wards, no more than 1 week after admission. Exclusion criteria will include neuromuscular diseases causing motor deficits, such as strokes, multiple sclerosis, amyotrophic lateral sclerosis, myasthenia gravis and Guillain-Barré syndrome. Patients will also be excluded in the event of the following: extubation less than 48 h after enrollment on the study; hemodynamic instability (norepinephrine > 0.5 μg/kg/min for arterial blood pressure > 60 mmHg); complications during the protocol such as pneumothorax, deep vein thrombosis or pulmonary embolism; Shilley catheter in the femoral vein; reintubation; delayed weaning (3 failed spontaneous ventilation tests); body mass index (BMI) > 35 kg/m^2^ [2]; or emergence of eschar in the calcaneus area during the protocol.

For sample selection, an assessor is going to conduct a daily search for potential trial participants using the hospital computer system. Then, electronic medical records will be reviewed by patient identification, medical diagnosis, and current medical condition in order to assess patients for eligibility. The next of kin to each eligible patient will be approached for study enrollment. Those who agreed to participate will be asked about the laterality of the patient and will be required to provide written consent.

### Interventions

Conventional (chest and motor) physical therapy is going to be performed by staff physical therapists who have at least 2 years of experience in the care of critically ill patients. All patients will receive 30-min physical therapy sessions twice daily (morning and afternoon). The protocol consists of passive diagonal movements based on the proprioceptive neuromuscular facilitation (PNF) stretching technique for the upper and lower extremities (two sets of 10 repetitions per set of each diagonal movement bilaterally) and manual bronchial hygiene techniques, such as chest compression-vibrations, resuscitation maneuvers with an Ambu® bag, Ambu, Ballerup, DK), and suction of secretions when necessary.

The intervention group (IG), in addition to conventional physical therapy, is going to undertake passive cycling exercise training for the lower extremities using a bedside cycle ergometer (Flexmotor; Cajumoro, São Paulo, SP, Brazil). For the cycling exercise, patients will be in the supine position with the head of the bed elevated to 30 °. Each patient will perform passive cycling movements at 20 rpm for 20 min once daily, 7 days a week, in the afternoon prior to conventional physical therapy, until extubation or day 7 of the protocol. The protocol will be discontinued if hemodynamic instability occurs (requiring norepinephrine >0.5 μg/kg/min for a mean arterial pressure (MAP) >60 mmHg), systolic blood pressure (SBP) >200 mmHg, heart rate <40 beats/min and persistent peripheral arterial saturation < 88 % or if performing tracheostomy is required.

Before and after each exercise session, the cycle ergometer will be cleaned following the cleaning procedures adopted in the ICU and the criteria established by the hospital infection control committee. Before starting the cycling exercise, all procedures will be briefly explained to the patient regardless of their level of consciousness or sedation. To minimize contact with the device, the heel region is going to be covered with sterile gauze and secured with adhesive tape so that the ankle joint will be close to a 90 ° angle. The passive cycling movements generate alternating extension and flexion of the knee and hip, bilaterally, for 20 consecutive minutes. All procedures will be performed under the supervision of one of the investigators.

In the two groups, arterial blood gas values will be recorded daily and the following parameters monitored during all sessions in order to monitor the safety of the technique: heart rate, respiratory rate, MAP, peripheral oxygen saturation, and ventilatory parameters.

After extubation or on day 7 of the protocol (whichever occurred first), all patients willreceive a second ultrasound examination (final assessment) and continue to receive conventional physical therapy until ICU discharge.

### Assessment

After completing the period of 24 to 48 h of MV, patients will be enrolled in the study and an ultrasound will be performed to assess the morphology and thickness of the knee extensors (dominant side) and excursion of the diaphragm muscle. The first ultrasound examination will be performed on the first day of patient participation in the study (initial assessment). A second examination will be performed on day 7 of the protocol or 24 h after extubation, whichever occurred first (final assessment). Both assessments will be performed by the same trained examiner, who will be blind to group assignment and data analysis.

With the patient lying supine, real-time B-mode ultrasound scanning will be performed using a 7.5 MHz linear-array transducer (SonoSite, Washington, DC, USA). The probe willbe coated with water-soluble transmission gel to provide acoustic contact without depressing the dermal surface [13]. First, the quadriceps muscle length will be identified and marked on the skin, and its midpoint determined. To ensure that the same region of the muscle will be scanned on subsequent sessions, a map of this region will be recorded and traced on a clear acetate sheet using a permanent marker. Bony prominences and birthmarks will also be included in the map, as well as the probe outline in order to ensure that the probe is held at the same angle relative to the frontal plane during all measurements [13]. Quadriceps muscle thickness will be determined on cross-sectional images by measuring the distance from the outer edge of the femur to the inner edge of the aponeurosis of the upper attachment of the rectus femoris muscle [14].

Ultrasound-based measurements of the vastus lateralis muscle architecture will be made on images obtained at sites corresponding to the points on the muscle belly of highest contractile muscle volume. The muscle length is going to be marked on the skin, its lower two thirds will be determined, and then a point will be marked on the vastus lateralis muscle belly. With the patient lying in the supine position with legs and knees extended, the hip unrotated and the ankle in a neutral position, the ultrasound transducer will be oriented along the axial plane of the vastus lateralis muscle belly. Axial-plane images of the vastus lateralis will then be acquired and muscle architecture parameters, such as fascicle length (FL), fascicle pennation angle (PA), and muscle thickness [15]. Finally, data on FL will be normalized to femur length for each patient.

Ultrasound measurement of the thickness of the diaphragm muscle will be conducted with patients lying in supine position. The probe will be coated in a water-soluble transmission gel to enable acoustic contact without depressing the surface of the skin. The probe will be positioned perpendicular to the diaphragm in the intercostal space over the tenth rib at the anteroaxillary line [16]. For image acquisition the probe will be coated in a water-soluble transmission gel to enable acoustic contact without depressing the surface of the skin. The probe will then be positioned perpendicular to the diaphragm and the image will be acquired for measurement of the thickness at the end of the inspiration [16].

Subjects will be positioned for assessment of diaphragm excursion lying down in the supine position. The probe will be coated in a water-soluble transmission gel to enable acoustic contact without depressing the surface of the skin. The probe will be positioned using the anatomic window for liver analysis between the medioclavicular line and the anterior axillary line, in the cranial direction. The probe will, therefore, be positioned medially, cranially and dorsally in such a way that the ultrasound beam transects the posterior third of the diaphragm [17, 18]. Inspiratory and expiratory diaphragmatic excursion images will be acquired with the ultrasound machine in M-mode. Inspiratory excursion will be defined as the vertical height measured from the base at the start of inspiration to the apex of inclination at the end of inspiration. Expiratory excursion will be defined as the vertical height from the apex of inspiration until the base returns [17, 18].

### Outcomes

The primary endpoint with respect to the efficacy of passive leg cycle exercise in preserving muscle morphology may be the difference in cross-sectional thickness of the dominant quadriceps muscle from initial to final assessment between groups. Secondary efficacy endpoints may include changes in muscle architecture parameters, such as vastus lateralis fascicle length, PA, muscle thickness, and diaphragm thickness and excursion. We will also assess length of ICU and hospital stay, duration of MV, successful extubation, and incidences of patient death. In addition, septic (requiring vasoactive drugs) and non-septic patients will be compared in terms of primary and secondary outcomes.

### Sample size calculation

The sample size calculation is based on a pilot study with 10 patients. To achieve an effect size of 1.2 standard errors between groups as cross-sectional thickness of the quadriceps muscle, significance level of 5 % and power of 85 % and 2 repeated measures (initial and final), the sample size estimated by the (WinPepi, http://www.brixtonhealth.com/pepi4windows.html) 11.43 statistical program was of 14 patients in each group.

### Randomization

Patients will be randomly assigned to receive either conventional physical therapy (conventional group) or exercise training intervention using a cycle ergometer associated with conventional physical therapy (intervention group). Randomization sequence is going to be created by using the website www.randomization.com, with a 1:1 allocation ratio using blocks of 10 participants.

To ensure the confidentiality of randomization sequence, the sequence will be generated by an assessor who has not participated in data collection or study design and will be contacted by telephone only after the participant has been included in the study and is ready to start the protocol.

### Statistical analysis

Continuous variables will be expressed as mean and standard deviation or standard error, or as median and interquartile range. Categorical variables will be expressed as absolute and relative frequencies. The Shapiro-Wilk test will be used to test the normality of distribution, and Levene’s test will be used to assess homogeneity of variance for all group comparisons. Student’s *t* test for independent samples will be used to compare means between groups, and the Mann–Whitney test will be used to compare medians between groups. Qualitative data will be analyzed using the chi-square test or Fisher’s exact test when at least 25 % of the cells exhibited the expected frequency <5. To evaluate the intra-group effects, the group and subgroups (septic versus non-septic) in changing muscle parameters, the model of Generalized Estimating Equations (GEE) will be performed with Bonferroni adjustment. Analysis of covariance (ANCOVA) will be used to control for confounding factors such as BMI, duration of protocol and duration of MV. Statistical analysis will be performed using SSPS, version 17.0 (SSPS Inc., Chicago, IL, USA). The level of significance is going to be set at 5 % (*p* ≤ 0.05).

## Discussion

It is becoming ever more widely recognized that physical training is an important component of caring for critical patients who require MV and one that can improve pulmonary and muscular function and functional independence, accelerating the recovery process and reducing the time spent on MV and in the ICU [19]. The potentially beneficial effects of early rehabilitation of critical patients who are immobile in bed are related to the theory of the calf muscle pump and muscle training. Physical exercise increases lower extremity muscle tone and, as a consequence, during muscle contractions there is increased ejection capacity, improving both venous return and muscle perfusion [20, 21].

Patients who are on MV are immobilized in bed and this can lead to muscle weakness rates of up to 25 %, can be associated with increased mortality and higher oxygen demand and can present challenges for weaning from ventilation [10, 22, 23]. ICU-acquired weakness can be caused by a range of factors, such as the inflammatory response and medications, and also because the cardiovascular system undergoes changes when patients spend prolonged periods lying down, including increased cardiovascular work and heart rate and changes to cardiac output, which in turn can lead to retention of fluid, causing edema [24].

Immobility in bed and the underlying critical disease lead to greater loss of muscle mass, particularly from the lower extremities, when compared with healthy individuals [2]. Delayed weaning can cause patients to develop pressure sores and worsens their physical fitness at the time of discharge from the ICU [10, 22–24]. In contrast, patients who are subject to intervention soon after admission to the ICU preserve a greater proportion of their physical capacity and functionality and achieve shorter hospital stays, although implementation of early rehabilitation in hospitals remains a challenge [25, 26].

In one controlled clinical trial [27], it was found that an early rehabilitation protocol was safe and easy to administer and led to shorter ICU stays and reduced expenditure when compared with patients given routine care. The members of the IG spent less time in bed, had shorter ICU stays and spent less time in hospital [27]. The authors also observed that 3 h of continuous passive rehabilitation using a cycle ergometer reduced fiber atrophy and protein loss, when compared with passive stretching for 5 min twice a day.

A study with healthy volunteers administered a cycling exercise test to exhaustion and then used ultrasound to assess quadriceps, finding that the PA and the thickness of the vastus lateralis muscle (VLMT) both increased [27]. The first randomized clinical trial to study the use and efficacy of a cycle ergometer with critical care patients demonstrated that one regular session of exercise daily was feasible and safe and should be administered early on in the ICU stay. The intervention improved functional capacity and muscle strength and brought forward hospital discharge in the patients who took part in that study [10].

In a study of critically ill patients published in *JAMA* recently, Puthucheary et al. [28] concluded that the loss of muscle mass occurred quickly and early during the first week of hospitalization. Risk stratification of patients with peripheral muscle wasting is vital to optimize the clinical management, including the implementation of kinesiotherapy, rehabilitation and other therapeutic interventions [29]. Recent attention has focused on the use of ultrasound to monitor the trajectory of muscle wasting in critically ill patients [30]. This had demonstrated clinical utility to evaluate the change in the architecture of peripheral skeletal muscles during critical illness. However, the technique still requires standardization of protocols in the intensive care environment [31].

It is now clear that the role of physiotherapy in the ICU and techniques employed are the subject of much research. A review of the recent literature showed that motor physiotherapy has proven beneficial for critical patients, reducing the time spent in the ICU and the hospital. Its effects on functional capacity were also positive, leading to the conclusion that early rehabilitation should be implemented in all ICUs [32].

## Trial status

Recruiting since May 2013.

## Abbreviations

ANCOVA: analysis of covariance
BMI: body mass index
CG: Conventional Group
FAPERGS: Fundação de Amparo à Pesquisa do Estado do Rio Grande do Sul
FIPE: Fundo de Incentivo à Pesquisa e Eventos
FL: length of fascicle
GEE: Generalized Estimating Equations
HCPA: Hospital de Clínicas de Porto Alegre
ICU: Intensive Care Unit
IG: Intervention Group
MAP: mean arterial pressure
MV: mechanical ventilation
PA: pennation angle of fascicles
PNF: proprioceptive neuromuscular facilitation
RCT: randomized controlled trial
rpm: revolutions per minute
SBP: systolic blood pressure
lUFRGS: Universidade Federal do Rio Grande do Sul
VLMT: thickness of vastus lateralis muscle
